# Rosuvastatin reduces nonalcoholic fatty liver disease in patients with chronic hepatitis C treated with α-interferon and ribavirin: Rosuvastatin reduces NAFLD in HCV patients

**Published:** 2011-02-01

**Authors:** Michele Malaguarnera, Marco Vacante, Cristina Russo, Maria Pia Gargante, Maria Giordano, Gaetano Bertino, Sergio Neri, Mariano Malaguarnera, Fabio Galvano, Giovanni Li Volti

**Affiliations:** 1 Department of Biochemistry, Medical Chemistry and Molecular Biology, University of Catania, Catania, Italy; 2 Department of Internal Medicine and Systemic diseases, University of Catania, Catania, Italy

**Keywords:** NAFLD, Ribavirin, Interferon, Hepatitis

## Abstract

**Background:**

Nonalcoholic fatty liver disease develops in patients with chronic hepatitis C. Interferon and ribavirin combination therapy is the standard treatment for chronic hepatitis C, but if present, NAFLD can reduce the virological response to anti-HCV therapies.

**Objectives:**

We determined whether the addition of rosuvastatin to interferon and ribavirin improves the sustained virological response (SVR) and reduces steatosis.

**Patients and Methods:**

This study was a prospective, randomized, open-label trial. Between January 2004 and December 2007, 65 patients with chronic hepatitis (27 women and 38 men, mean age 48 years) aged 32-63 years (median 46 years) were consecutively enrolled. Patients were randomly assigned to receive leukocyte interferon alpha (3 MIU 3 times per week) plus ribavirin (1200 mg per day) for 12 months or interferon alpha and ribavirin at the same dosages plus rosuvastatin (5 mg per day). The primary endpoints were measurements in SVR, liver enzyme, cholesterol, triglyceride, CRP, glucose, and insulin levels; and Homa-IR, fibrosis, and steatosis scores.

**Results:**

After 12 months of treatment, we observed a significant improvement in SVR in 51% of patients who received interferon plus ribavirin plus rosuvastatin compared with 18% of relapsers (OR 1.52; 95% CI = 0.41-5.64; RR 1.13). There were 23 responders (69%) and 10 nonresponders (30%) (OR 1.38; 95% CI = 0.49-16.5; RR 1.11). When comparing interferon plus ribavirin group vs interferon plus ribavirin and rosuvastatin group after 12 months, we observed a significant difference in AST (85.70 vs.106.5.00 IU/ml) (OR 1.2; 95% CI= 0.29-4.94; RR 1.04; p<0.001), ALT (81.80 vs. 126.2 IU/ml) (OR 1.2; 95% CI = 0.29-4.94; RR 1.04; p < 0.001), LDL-cholesterol (0.01 vs. 0.60 mmol/l) (OR 14; 95% CI = 3.98-49.16; p RR 2.96; < 0.001), triglycerides (0.17 vs. 0.2 mmol/l) (OR 20; 95% CI = 4.94-80.89; RR 5.38; p < 0.05), and Viremia (1.8 vs. 2.48 UI/ml, p < 0.05). Mean fibrosis score decreased 0.10 vs. 0.50 (OR 4.5; 95% CI = 0.89-22.66; RR 1.5; p < 0.05), and mean steatosis score declined 0.30 vs. 0.50 (OR 11.2; CI = 2.88-43.53; RR 2.75; p < 0.001).

**Conclusions:**

In HCV patients with NAFLD, the addition of rosuvastatin to interferon and ribavirin significantly reduces viremia, steatosis, and fibrosis without causing side effects.

## Background

Nonalcoholic fatty liver disease (NAFLD) affects 40% to 85% of patients with chronic hepatitis C [1][2]. This correlation appears to depend on host and viral factors [3]. Recent studies have suggested that interactions between hepatitis C virus (HCV) core protein and lipid droplets are required for the HCV infection cycle. In infected cells, the HCV core protein associates with the surface of lipid droplets. This interaction also appears to be linked directly to virus-induced steatosis, which entails the deposition of triglycerides in the liver and accelerates the progression of fibrosis in patients with chronic hepatitis C [4]. Many clinical studies have reported that virus-induced steatosis is significantly more severe in those with HCV genotype 3 than with other genotypes [5]. Some virus that is recovered from the blood of infected patients binds to host lipoprotein particles that contain apolipoprotein B100 (apoB100) and apolipoprotein E (apo E), the predominant proteins in VLDL. These viral RNA host lipoprotein complexes are called lipo-viro-particles (LVPs) [6]. Recent reports indicate that the nascent virus and host lipoprotein form LVPs in the endoplasmic reticulum of hepatoma cells, which is necessary for release of the virus. The formation of LVPs might favor viral infectivity or persistence in the host and can interfere with the normal secretion or uptake of host cell lipoproteins and mediate the pathology of persistent viral infection [7]. Consequently, HCV-infected patients, whether they have NAFLD, develop a chronic and progressive disease that sometimes responds poorly to treatments [8][9]. Statins inhibit hepatitis C viral RNA replication in vitro with nearly the same efficacy as the most potent clinical therapeutics [10]. Ikeda et al. used a genome-length HCV RNA replication system to evaluate the anti-HCV activity of statins and their effects in combination with interferon α. In the study by Ikeda et al. five statins were examined: atorvastatin, fluvastatin, pravastatin, simvastatin, and lovastatin. Atorvastatin, fluvastatin, and simvastatin had stronger anti-HCV activity, and pravastatin exhibited no such activity, although it inhibited HMG-CoA reductase. Because fluvastatin had the most robust anti-HCV activity, it was examined in combination with interferon-α, demonstrating synergistic inhibitory effects on HCV RNA replication [10]. Recently, another study demonstrated that fluvastatin has anti-hepatitis C virus activity by inhibiting the geranylgeranylation of cellular proteins synergistically with interferon-α [11]. Nevertheless, statins upregulated low-density lipoprotein (LDL) receptor, which is required for HCV cell entry, and the closely related scavenger receptors SRBI and CD36. Aizaki et al. reported that lovastatin, an HMG-CoA reductase inhibitor, impeded HCV RNA replication in HCV replicon-harboring cells [11]. Ikeda et al. also demonstrated that lovastatin suppressed HCV RNA replication. These reports suggest that the anti-HCV activity of lovastatin results from the inhibition of protein geranylgeranylation rather than of cholesterol synthesis. In vivo LDL levels are a prognostic indicator of sustained viral response to interferon in patients with HCV infection, suggesting that lipid-lowering agents favor HCV entry into hepatocytes, effecting higher viral replication [12]. Moreover, clinicians have been reluctant to use statins as a treatment for human hepatitis C trials due to their potential hepatotoxicity in chronic liver disease [13][14]. These concerns were addressed by a recent trial, which demonstrated that high doses of pravastatin were safe and well tolerated [15]. Of the statins, rosuvastatin is a hydrophilic statin that potentially has limited access to nonhepatic cells due to low passive diffusion and undergoes robust hepatic cell uptake via selective organic anion transport.

## Objectives

The aim of this study was to determine whether the addition of rosuvastatin to interferon and ribavirin increases the sustained virological response (SVR) and if it reduces steatosis by improving hepatic histology.

## Patients and Methods

### Study Design

This 12-month, randomized, placebo-controlled trial was performed per the Declaration of Helsinki [16] and was approved by the local ethics committee. It was conducted in the Department of Internal Medicine, Cannizzaro Hospital, University of Catania, Catania, Italy. All patients provided written informed consent before participating in the study.Eligible patients were randomly assigned equally to one ofe two study treatments by a computer-generated table of random numbers, allocated in our central unit. They were divided into 2 groups (A and B) and stratified by HCV genotype (1 vs. others) and viral load (≤600,000 IU/ml vs. >600,000 IU/ml). Group A received leukocyte interferon alpha 3 MIU (Alfaferone; Alfa Wasserman Italy) intramuscularly 3 times per week for 12 months plus daily oral ribavirin (800 mg for body weight below 60 kg, 1000 mg between 60 and 75 kg, and 1200 mg above 75 kg). Group B received leukocyte interferon alpha and ribavirin at the same dosages, route, and duration plus rosuvastatin 5 mg per day. Patients were evaluated before treatment and 2 weeks, 6 months, and 12 months after initiation of the therapy. A follow-up evaluation was performed 6 months after the end of the planned treatment. A medical interview and physical examination were conducted for all patients before therapy was begun.

### Patients

Between January 2004 and December 2007, 65 patients with chronic hepatitis (27 women and 38 men, mean age 48 years) aged 32-63 years (median 46 years) were consecutively enrolled (32 patients in the interferon and ribavirin group; 33 patients in the interferon and ribavirin plus rosuvastatin group) (Table 1-Table2). The patients had to meet the following inclusion criteria: alanine aminotransferases (ALT) levels greater than 1.5-fold higher than the upper limit of normal, the presence of anti-HCV antibodies in the serum, HCV-RNA >1000 copies/ml, and histological changes on the liver biopsy. Exclusion criteria were: positivity for serum hepatitis B surface antigen, positive serum HIV antibody test, negativity for HCV antibodies, alcoholic liver disease (daily alcohol consumption <20 g/day), and diabetes. The presence of other causes of hepatopathy, decompensated cirrhosis, pregnancy, formal contraindications for interferon or ribavirin therapy (such as hemoglobinopathies, cardiopathy, hemocromatosis, diabetes mellitus, autoimmune diseases, major depression and any other severe psychiatric pathological condition) and use of an illicit treatment or drug that might have influenced serum lipid levels within the last 12 months were causes for exclusion. Baseline demographics and histological findings on the liver biopsy were similar between the 2 treatment groups. The mean times since chronic hepatitis C infection were comparable. The most frequent viral genotype was 1 b. Baseline viremia was similar in the 2 groups. ALT, AST, prothrombin time, total cholesterol, triglycerides, fasting plasma glucose, CRP, insulin, and HOMA-IR did not differ between groups.

**Table 1:** Patient characteristics at liver biopsy

**Parameter **(Normal Value)	**Group A **(IFNα and Ribavirin)	**Group B** (IFNα and Ribavirin+Rosuvastatin)	**p-value**
**N**	32	33	
**Age **(year)	47.4 ± 5.2	47.8 ± 5.9	NS
**Gender **(M/F)	18/14	20/13	NS
**Time since exposure **(year)	5.08 ± 3.4	5.12 ± 3.6	NS
**BMI **(Kg/m(2))	25.8 ± 3.6	26.8 ± 3.0	NS
**Probable exposure **(No of patients)			
**Blood transfusion**	14	13	NS
**Infected needle**	6	7	NS
**Healthcare environment**	2	2	NS
**Other/unknown**	10	11	NS
**Genotype**			
**1a**	3	3	
**1b**	23	23	
**2a**	2	1	
**3a**	4	6	
**Laboratory parameter**			
**Glucose **(mmol/L) (normal: 3.9-6.4)	5.97 ± 0.48	6.01 ± 0.54	NS
**Insulin **(mIU/L) (normal: <19)	17.0 ± 5.1	17.6 ± 5.0	NS
**HOMA-IR**	4.51 ± 0.24	4.70 ± 0.32	NS
**AST **(IU/L) (normal: 15-50)	167 ± 34	164 ± 40	NS
**ALT **(IU/L) (normal: 15-50)	169 ± 47	170 ± 38	NS
**Cholesterol** (mmol/l) (normal: 0-5.2)	5.11 ± 0.66	5.12 ± 0.64	NS
**Triglycerides **(mmol/l) (normal: 0.3-2.8)	2.36 ± 0.50	2.28 ± 0.58	NS
**Viremia **(106 copies/ml)	5.04 ± 3.86	5.00 ± 3.44	NS
**CRP **(mg/dl) (normal: <1.0)	3.21 ± 0.62	3.31 ± 0.57	NS

### Laboratory exams

A complete routine chemical workup, measuring red cell count, hemoglobin, white cell count, platelet, prothrombin time, fasting plasma glucose, insulin, CRP, blood urea nitrogen, serum creatinine, bilirubin, alanine aminotransferase, aspartate aminotransferase, alkaline phosphatase, γ-glutamil transpeptidase, and creatin phosphokinase levels, was performed at every medical visit. We also measured total cholesterol, HDL-cholesterol, low-density lipoprotein cholesterol (LDL-C), and triglycerides at baseline, 2 weeks, 6 and 12 months, and the follow-up visit. An enzymatic assay was used to determine serum total cholesterol and triglycerides levels (Hitachi 704 analyser-twin TG/CHO reactive, Boehringer Mannheim automated analysis, Germany). HDL-C was measured by another enzymatic assay after precipitating lipoproteins that contained apolipoprotein B with phosphotungstic acid/magnesium chloride. LDL-C levels were calculated using Friedewald's method [17]. Serum-sensitive C-reactive protein (CRP) was measured by particle-enhanced immunoturbidimetric assay (detection limit 0.04 mg/L; Roche Diagnostics Mannheim Germany). Anti-HCV antibodies were measured using a second-generation ELISA (Ortho-Diagnostic Systems, Raritan NJ, USA), and positive samples were been confirmed by immunoblot (RIBA; Chiron Corporation, Emeryville, CA-USA). We measured serum HCV-RNA levels by quantitative (Cobas Amplicor HCV Monitor test, version 2.0) and qualitative tests (Cobas Amplicor HCV test, version 2.0; limit of detection 50 IU/ml). HCV genotypes and subtypes were determined by a modified specific line probe assay (Inno-LiPA system; Innogenetics NV, Zwijnaarde, Belgium), as described by Stuyver et al [18]. The HCV genotypes were designated based on the nomenclature proposed by Simmonds [19].

**Table 2:** Baseline characteristics of subjects at outcome, 12 months, and follow-up

**Variable**	**Group A**(interferon α+Ribavirin) **n=32**			**Group B** (interferon α+Ribavirin+Rosuvastatin) **n=33**			**P-value Group A vs. Group B **b	
	Before treatment	After treatment	Follow-up	Before treatment	After treatment	Follow-up	After treatment	Follow-up
**AST **(IU/ml) a	146.1 ± 49.8	60.4 ± 38.0 (p < 0.01)	78.2 ± 38.6 (p < 0.001)	145.0 ± 46.8	38.5 ± 30.2 (p < 0.001)	50.0 ± 34.1 (p < 0.001)	p < 0.001	p < 0.001
**ALT **(IU/ml) a	160.0 ± 49.4	78.2 ± 40.2 (p < 0.001)	84.6 ± 46.1 (p < 0.001)	184.0 ± 49.2	57.8 ± 21.4 (p < 0.001)	78.4 ± 28.8 (p < 0.001)	p < 0.001	NS a
**Total Cholesterol **(mmol/L)	5.11 ± 0.8	5.0 ± 0.9 (NS ) a	5.0 ± 0.7 (NS ) a	5.2 ± 0.7	4.6 ± 0.8 (p < 0.05)	4.9 ± 0.9 (NS) a	NS a	NS a
**HDL **(mmol/L) a	1.06 ± 0.8	1.07 ± 0.8 (NS) a	1.04 ± 0.9 (NS) a	1.08 ± 0.8	1.1 ± 0.2 (NS) a	1.05 ± 0.7 (NS) a	NS a	NS a
**LDL **(mmol/L) a	2.50 ± 0.73	2.51 ± 0.64 (NS) a	2.5 ± 0.6 (NS) a	2.6 ± 0.78	2.0 ± 0.8 (p < 0.05)	2.2 ± 0.7 (p < 0.05)	p < 0.001	NS a
**Triglycerides **(mmol/L)	2.24 ± 0.71	2.41 ± 0.61 (NS) a	2.8 ± 0.74 (NS) a	2.2 ± 0.8	2.0 ± 0.4 (NS) a	2.1 ± 0.6 (NS) a	p < 0.05	p < 0.001
**Viremia **(10(6) copies/ml)	3.24 ± 1.22	1.44 ± 0.87 (p < 0.001)	2.10 ± 0.98 (p < 0.001)	3.44 ± 1.18	0.96 ± 0.70 (p < 0.001)	1.33 ± 0.87 (p < 0.001)	p < 0.05	p < 0.001
**CRP **(mg/dl) a	3.24 ± 0.56	7.04 ± 0.61 (p < 0.001)	5.21 ± 0.44 (p < 0.001)	3.43 ± 0.47	4.01 ± 0.61 (p < 0.001)	1.96 ± 0.32 (p < 0.001)	p < 0.001	NS a
**Plasma Glucose **(mmol/L)	5.87 ± 0.67	6.18 ± 0.67 (NS) a	5.91 ± 0.71 (NS) a	5.96 ± 0.71	5.84 ± 0.56 (NS) a	5.47 ± 0.51 (p < 0.05)	p < 0.05	p < 0.05
**Insulin** (mIU/ml)	17.06 ± 5.0	19.6 ± 4.8 (p < 0.05)	17.9 ± 6.2 (NS) a	17.44 ± 5.1	16.9 ± 5.4 (NS) a	16.0 ± 5.2 (NS) a	P < 0.05	NS a
**HOMA-IR**	3.14 ± 0.15	5.38 ± 0.14 (p < 0.001)	4.02 ± 0.18 (p < 0.001)	4.56 ± 0.16	4.38 ± 0.14 (p < 0.001)	3.88 ± 0.12 (p < 0.001)	p < 0.001	p < 0.001

^a^ NS: not significant; AST: Aspartate Amino Transferase; ALT: Alanine Amino Transferase; HDL: High-Density Cholesterol Lipoprotein; LDL: Low-Density Cholesterol Lipoprotein; CRP: C-Reactiv

^b^ There were no significant differences between groups at baseline.

### Histology

Liver biopsy was performed in the 6 months before the initiation of therapy and 6 months after the end of treatment by modified Menghini technique. The specimen was fixed in 4% neutral formaldehyde solution for routine histological processing and evaluation. The Knodell and Ishiak Histological activity index (HAI) score was used to assess the histological grade of the disease [20]. Steatosis was graded on a scale from 0 to 4, based on the percentage of cells with fat-0 = none, 0.5 (trace) to < 5%; 1 = 5% to < 25%; 2 = 25% to < 50%; 3 = 50% to < 75%; and 4 = 75% to 100%. The pathologist was blinded to the treatment arms. The fibrosis stages were: 0 = no fibrosis, 1 = portal fibrosis without septa; 2 = portal fibrosis with rare septa; 3 = numerous septa without cirrhosis; and 4 = cirrhosis. The degree of steatosis was assessed based on the percentage of hepatocytes that contained fat droplets.

### Efficacy and safety assessment

All enrolled patients were included in the intention-to-treat efficacy analysis (ITT), and patients who received at least 1 dose of interferon-α plus ribavirin were included in the safety analysis. Data were analyzed using an intention-to-treat principle. We considered patients to be "sustained virological responders" (SVRs) when HCV RNA was not detected (< 50 IU/ml) in serum at the end of the follow-up period. Relapse was defined as undetectable HCV-RNA levels at the end of treatment but detectable levels during the follow-up period. Adverse events were assessed by interviews and laboratory and clinical examinations during treatment. They were graded as mild, moderate, and severe based on WHO score. The treatment was halted if severe events occurred, such as hematological toxicity, hepatic failure, and lack of compliance. In moderate and mild cases of adverse effects, the dose was reduced 50%, until resolution of the event, at which point a full dose was restarted.

### Statistical analysis

Results are expressed as means ± standard deviations. Comparisons of quantitative data were made by Student's t-test or Mann-Whitney test. Qualitative data were analyzed by chi-square test. A p-value < 0.05 indicated a statistically significant difference. All data management and statistical calculations were performed using SPSS 15.0 (Chicago, IL, USA).

## Results

In the group that was treated with interferon plus ribavirin, AST (p<0.001) and ALT (p < 0.001) levels decreased significantly in 46% of patients after 12 months. CRP levels (p < 0.001), insulin (p < 0.05), HOMA-IR (p < 0.001), and viremia fell significantly (p < 0.001). Moreover, mean inflammatory score (p < 0.05) and status scores declined (p < 0.05). The same results were observed at the follow-up. In the group that was treated with interferon plus ribavirin and rosuvastatin, AST (p < 0.001) and ALT (p < 0.001) levels decreased in 54% of patients after 12 months. Total cholesterol (p < 0.05), LDL-cholesterol (p < 0.05), CRP (p < 0.001), HOMA-IR (p < 0.001), and viremia also fell significantly (p < 0.001). At the follow-up time we observed a decrease in AST (p < 0.001), ALT (p < 0.001), LDL cholesterol (p < 0.05), CRP (p < 0.001), HOMA-IR (p < 0.001), and plasma glucose (p < 0.05) (Table 2). Mean inflammatory score (p < 0.001), mean fibrosis score (p < 0.05), steatosis score (p < 0.001) also declined (Table 3). When comparing interferon plus ribavirin group vs interferon plus ribavirin and rosuvastatin group after 12 months, we observed a significant difference as concerns the decrease in AST 85.70 vs. 106.5.00 IU/ml (OR 1.2; 95% CI= 0.29-4.94; RR 1.04; p < 0.001), ALT 81.80 vs. 126.2 IU/ml (OR 1.2; 95% CI= 0.29-4.94; RR 1.04; p < 0.001), LDL cholesterol 0.01 vs. 0.60 mmol/l (OR 14; 95% CI= 3.98-49.16; RR 2.96; p < 0.001), Triglycerides 0.17 vs. 0.2 mmol/l (OR 20; 95% CI= 4.94-80.89; RR 5.38; p < 0.05). Viremia values decreased significantly (p < 0.05) (1.8 vs. 2.48 UI/ml). Therefore we observed a significant difference as concerns the increase in CRP 7.04 vs. 4.01 mg/dl (p < 0.001), plasma glucose 6.18 vs. 5.84 mmol/l (p < 0.05), HOMA-IR 5.38 vs. 4.38 (p < 0.001), insulin 19.6 vs. 16.9 mIU/L (p < 0.05). After 12 months, after treatment with interferon plus ribavirin plus rosuvastatin, SVR improved in 17 patients versus 13 patients who were treated with interferon plus ribavirin (51% vs. 40%), in 6 versus 7 relapsers, respectively (18% vs. 21%) (OR 1.52; 95% CI= 0.41-5.64; RR 1.13; p < 0.05), in 23 versus 20 responders, respectively (69% vs. 62%), and in 10 versus 12 nonresponders, respectively (30% vs. 37%) (OR 1.38; 95% CI= 0.49-16.5; RR 1.11; p < 0.001). When comparing interferon plus ribavirin group vs interferon plus ribavirin and rosuvastatin group after 12 months, we observed a significant difference as concerns the decrease in mean fibrosis score 0.10 vs. 0.50 (OR 4.5; 95% CI= 0.89-22.66; RR 1.5; p < 0.05), and mean steatosis score 0.30 vs. 0.50 (OR 11.2; CI= 2.88-43.53; RR 2.75; p < 0.001). Baseline alanine aminotransferase values, normal fasting glucose, and rosuvastatin treatment were predictors of SVR in the univariate analysis, but no predictors were independently associated with SVR in the multivariate analysis.

**Table 3:** Liver histological features

**Variable**	**Group A** (IFN α+Ribavirin) **n=32**			**Group B** (IFN α+Ribavirin+Rosuvastatin) **n=33**			**p-value Group A vs. Group B**	
	**Before treatment**	**After treatment**	**P-value**	**Before treatment**	**After treatment**	**P-value**	**Baseline**	**After treatment**
**Mean Inflammation Score **(range)	7.4 ± 2.6 (5-11)	6.0 ± 2.4(4-10)	< 0.05	7.7 ± 2.8 (5-11)	6.0 ± 2.0 (3-9)	< 0.001	NS	< 0.05
**Mean Fibrosis Score **(range)	1.6 ± 0.3 (1-4)	1.5 ± 08 (1-4)	NS	1.5 ± 0.4 (1-4)	1.0 ± 0.8 (0-4)	< 0.05	NS	NS
**Steatosis Score**(range)	2.3 ± 0.4 (1-4)	2.0 ± 0.4 (1-4)	< 0.05	1.9 ± 0.3 (1-4)	1.4 ± 0.5 (0-4)	< 0.001	NS	< 0.001

### Adverse events

No serious adverse events (World Health Organization grade 3 or 4) were reported in the 2 groups. Six patients who were treated with interferon plus ribavirin and 2 in the other group experienced mild psychological disorders, such as anxiety, irritability, and depression. Median hemoglobin concentration fell significantly during the first 3 months of treatment in both groups and stabilized for 3 months, returning to near-baseline values within 3 months after the end of the treatment. Notably, hemoglobin values decreased to a greater extent in the interferon plus ribavirin group. In patients who were treated with interferon plus ribavarin plus rosuvastatin, median hemoglobin concentration fell from 13.1 g/dl (range 11.2-14.0 g/dl) to 11.3 (range 10.4-14.0 g/dl) at the end of therapy. The patients who were treated with interferon plus ribavirin experienced a decrease in median hemoglobin concentration from 13.0 g/dl (range 11.4-15.1) to 10.5 g/dl (range 10.0-12.4 g/dl) at the end of therapy. The interferon plus ribavirin group showed a significant decrease in white cell blood count. Platelet counts did not change significantly in either group. Furthermore, both groups experienced anorexia (12% in interferon plus ribavirin patients and 16% in the interferon plus ribavirin plus rosuvastatin group), nausea (20% and 24%, respectively), weight loss (14% and 5%, respectively), headache (44% and 48%, respectively), fatigue (44% and 55%, respectively), myalgia (30% and 55%, respectively), musculoskeletal pain (30% and 42%, respectively), irritability (18% and 22%, respectively), hypertriglyceridemia (34% and 18%, respectively), hypercholesterolemia (24% and 8%, respectively), and hyperglycemia (12% and 4%, respectively). Sixty-two percent of patients adhered to their medication dose and duration of therapy.

## Discussion

Effective management of chronic HCV infection is critical [21]. The goal of treatment for chronic HCV infection is sustained virological response (SVR), accompanied by improvements in liver damage. The potential benefits of SVR are decreased infectivity, prevention of liver damage, and improved necroinflammation. Long term, SVR may decrease one's risk of developing cirrhosis, decompensation, and HCC; prolong survival; and improve quality of life [22][23][24][25]. The current standard of hepatitis C treatment is the combination of pegylated interferon-α (Peg- interferon-α) with ribavirin. This regimen has been successful in patients with HCV genotype 2 and 3 infections, effecting HCV eradication rates of 75% to 90%. However, it is much less effective in patients with genotype 1 and 4 infections, yielding eradication rates that range from 45% to 52%. Recent studies on HCV replicons have implicated statins as therapeutics for chronic HCV infection. The effect of 3-hydroxy-3-methyl-glutaryl-coenzyme A reductase inhibitors on HCV replication in human subjects has been examined prospectively in studies, with contrasting results. Some studies noted no reduction in HCV RNA titers relative to baseline levels [26][27][28][29], whereas other studies observed an unsustained, non-dose-related reduction in HCV RNA titers [29][30].

We demonstrated significant beneficial effects of oral rosuvastatin, added to interferon alpha and ribavirin, on lipid metabolism, inflammation markers, status, and fibrosis. The effects persisted after 12 months and at the follow-up visit, and patients with HCV tolerated rosuvastatin well. Moreover, viremia declined in both groups, but rosuvastatin had a greater effect when added to interferon and ribavirin compared with interferon plus ribavirin alone. We hypothesize that because status negatively influences the response rate to antiviral treatment, as shown in large clinical trials, the management of status in chronic hepatitis C patients can improve the success of pharmacological interventions [31]. The combination of rosuvastatin, interferon alpha, and ribavirin appears to be more efficacious than interferon-α plus ribavirin in the treatment of ongoing NAFLD in patients with chronic hepatitis C. Rosuvastatin improves lipid profiles, hepatic parameters, and the histology of HCV. In patients with HCV, rosuvastatin decreases total cholesterol, LDL, and triglyceride levels and reduces CRP, status, fibrosis, and inflammation [32]. Moreover, rosuvastatin limits the effects of cytokines, improving insulin resistance. Interleukin 1 and TNF, produced by monocytes and macrophages after stimulation with interferon, induce inflammation and increase serum triglyceride levels by stimulating hepatic lipogenesis, which promotes ongoing infections and progressive damage [33][34][35][36]. Thus, the antagonism of rosuvastatin against lipid metabolism and fat accumulation is a new therapeutic mechanism that can be used to improve antiviral therapies.

Statins might have indirect antiviral effects through mechanisms that are unrelated to lipid metabolism, suggesting that statins have a place in the management of HCV patients. Nevertheless, they carry a risk of elevated asymptomatic liver enzymes and hepatotoxicity, necessitating cautious use in patients with fatty liver disease and hepatitis. In conclusion, our results suggest that statins, such as rosuvastatin, are good adjuncts to interferon therapy in patients with NAFLD and chronic hepatitis C. We hypothesize that rosuvastatin improves responses to treatments that reduce persistent inflammation of the liver. Limitations of our study include its relatively small sample size and its open-label design. Further, no subanalysis was performed for various HCV genotypes, due to the prevalence of genotype 1. Moreover, we used interferon rather than peginterferon, because we noted that interferon is well tolerated and has a shorter half-life and that fewer patients dropped out of the study. There are several concerns with regard to the safety of peginterferon, especially in patients with cirrhosis, who experience a high occurrence of adverse effects. As a consequence, they have to discontinue the treatment. Future trials should assess the increased risk of side effects of statins in these patients and the true efficacy of the combination therapy. Nevertheless, our results suggest that rosuvastatin is an efficacious agent that can be combined with interferon in patients with chronic hepatitis C and ongoing NAFLD.
